# Overview of pharmacodynamical research of traditional Chinese medicine on hyperuricemic nephropathy: from the perspective of dual-regulatory effect on the intestines and kidneys

**DOI:** 10.3389/fphar.2025.1517047

**Published:** 2025-04-08

**Authors:** Ting Wang, Li Li, Li Liu, Ruirong Tan, Qinxuan Wu, Xin Zhu, Hua Hua, Ying Dai, Huan Li, Jiuzhou Mao, Junning Zhao, Zhujun Yin

**Affiliations:** ^1^ Country School of Pharmacy, Southwest Medical University, Luzhou, China; ^2^ Sichuan Academy of Chinese Medicine Sciences, Institute of Pharmacology & Toxicology of Chinese Materia Medica, Translational Chinese Medicine Key Laboratory of Sichuan Province, Sichuan Engineering Technology Research Center of Genuine Regional Drug, Engineering Research Center for Formation Principle and Quality Evaluation of Genuine Medicinal Materials in Sichuan Province, Chengdu, China; ^3^ Sichuan Institute for Translational Chinese Medicine, Translational Chinese Medicine Key Laboratory of Sichuan Province, Chengdu, China; ^4^ Changsha Medical University, Hunan Provincial Key Laboratory of the Research and Development of Novel Pharmaceutical Preparations, The “Double-First Class” Application Characteristic Discipline of Hunan Province (Pharmaceutical Science), Changsha, China; ^5^ Sichuan Acupuncture and Moxibustion School, Chengdu, China

**Keywords:** hyperuricemic nephropathy, gut microbiota, urate transporters, immuno-inflammatory response, traditional Chinese medicine

## Abstract

Uncontrolled hyperuricemia contributes to chronic kidney disease, characterized by renal inflammatory cell infiltration and tubulointerstitial fibrosis, eventually leading to renal failure. In addition to liver and kidney, the intestine tract plays a vital role in the development and progression of hyperuricemia and hyperuricemic nephropathy (HN) through various mechanisms. The conventional therapeutic strategy for HN is uric acid-lowering therapy (ULT) and renal protection; however, unsatisfactory results are often obtained in clinical practice. Growing evidence has demonstrated that traditional Chinese medicines (TCMs) achieve an anti-HN effect by modulating multiple targets and approaches with fewer side effects. Therefore, this paper reviews the pathogenesis of HN, including the role of soluble and insoluble urates in kidney and intestine, and the role of intestinal tract in the progression of HN. Meanwhile, the recent advancements in TCMs for the treatment of HN are summarized and analyzed, with a focus on their modulation of intestinal flora and metabolites, urate-related transporters, immuno-inflammation and barrier function in the intestines. Notably, for the first time, we propose the perspective that TCMs treat HN through a dual-regulatory effect on the intestines and kidneys. Additionally, the problems existing in current research and the feasible research strategies combined with emerging technologies such as fermentation and nanotechnology are discussed, thus providing novel ideas for HN management.

## 1 Introduction

Hyperuricemia (HUA) is one of the most common metabolic diseases, resulting from the metabolic disorder of purine and uric acid. Currently, the prevalence of hyperuricemia is steadily rising, ranging from 2.6%–36% across the globe (Jiang J. et al., 2023), and is especially higher in the countries with high-purine and high-fructose consumption. It was estimated that the prevalence of hyperuricemia among Chinese population was 14.0% in 2018–2019, with the younger population suffering more, as deduced from two nationally representative cross-sectional investigations (Zhang M. et al., 2022). Chronic hyperuricemia might contribute to renal dysfunction, which can further develop to hyperuricemia nephropathy (or gouty nephropathy) and increases the risk of chronic kidney disease (CKD). It has been proposed that hyperuricemia nephropathy is a secondary inflammatory nephropathy induced by the deposition of monosodium urate (MSU) crystals in the distal collecting duct and the medullary interstitium (Kang, 2014). Autopsy examinations have confirmed that 75%–99% of gouty subjects exhibit prominent alterations in renal histopathology, including MSU deposits, arteriolosclerosis, glomerulosclerosis, and tubulointerstitial fibrosis (Linnane et al., 1981). Moreover, a large amount of literature has documented that asymptomatic hyperuricemia also increases the progression of CKD, cardiovascular disease, hypertension, diabetes mellitus, and metabolic syndrome (Aktas et al., 2022; Gaubert et al., 2020). Previous studies have demonstrated that chronic hyperuricemia induces glomerulosclerosis and tubulointerstitial fibrosis via sustained inflammation, endothelial dysfunction, oxidative stress amplification, and dysregulated renin-angiotensin system (RAS) activation, ultimately culminating in progressive renal dysfunction (Du et al., 2024). The deposition of excess urate in the renal tubules or extrarenal tissues triggers multiple intracellular signaling pathways, such as MAPK, PI3K/Akt, and NF-κB, which promote oxidative stress and inflammatory responses (Li K. et al., 2023). Additionally, MSU act as injury-associated molecular patterns, initiating cellular pyroptosis via the activation of the NLRP3 inflammasome signaling pathway. This results in the massive release of active IL-1β, which further amplifies the renal inflammatory cascade and accelerates the progression of renal fibrosis (Hu et al., 2022).

Although it is debatable whether elevated uric acid or MSU deposits are the main cause of hyperuricemia nephropathy, uric acid-lowering therapy is recommended to slow the progression of CKD and other complications (Fang et al., 2023). Given that hyperuricemia is a prerequisite for the occurrence of uric acid nephropathy, understanding why hyperuricemia occurs is essential for recognizing the pathogenesis and developing treatments for hyperuricemia nephropathy. Uric acid, the final product of purine nucleotide metabolism, can contribute to hyperuricemia when there is excessive production or abnormal excretion. A high-purine diet or other dietary factors [such as alcohol (Choi et al., 2004)] and high-fructose beverage consumption (Huang Y. et al., 2023; Zhang et al., 2020) increase serum uric acid levels and the incidence of gout flares. However, the uric acid from dietary sources only accounts for 20% of the total uric acid pool in the human body. Approximately 80% of serum uric acid is derived from the metabolism and transport of endogenous uric acid. The liver, as the primary organ responsible for purine metabolism, orchestrates the catabolism of nucleic acids and the *de novo* synthesis of purine nucleotides from amino acids and other precursors, accounting for approximately 70% of endogenous uric acid production (Sharaf El Din et al., 2017). Notably, abnormalities in glycolipid metabolism significantly activate xanthine oxidase, which accelerates uric acid biosynthesis and promotes the development of hyperuricaemia (Johnson et al., 2011). It has been well-documented that about two-thirds of uric acid is excreted by the kidneys, while the remaining third is excreted by the intestines. It is worth noting that the intestinal tract plays a vital role in purine and uric acid metabolism, influenced by the urate transporters, diversity of microbiome and its sensitive mechanisms for dealing with harmful insults. Recent evidence suggested that the imbalance of the intestinal flora accelerated the progression of kidney diseases, including hyperuricemia nephropathy, diabetic nephropathy and septic nephropathy. Moreover, supplement of prebiotics and probiotics have been confirmed to ameliorate the renal dysfunction, further delaying the occurrence of CKD (Cao J. et al., 2022; García-Arroyo et al., 2018). However, the specific interplay between renal and intestinal factors and the underlying mechanisms of hyperuricemia nephropathy remain poorly understood.

Whether hypouricemic medication targeting the proteins that govern the metabolism of purines and uric acid have renoprotective potential for individuals suffering from hyperuricemia and gout depends. Due to the potential for renal impairment, the clinical application of allopurinol is cautious (Badve et al., 2020), while probenecid is contraindicated when CrCL is <60 mL/min (Kang, 2014). Unlike allopurinol, dosing adjustments are not needed for patients with renal impairment when using febuxostat, a nonpurine selective inhibitor of xanthine oxidase, as the hypouricemic agent (Becker et al., 2005). Compared to febuxostat, benzbromarone (25 mg daily) has been confirmed to be more potent in hyperuricemia patients with eGFR ≥ 70 mL/min/1.73 m^2^ (Liu et al., 2022). Additionally, one of the common complications of probenecid and benzbromarone is the deposition of urates or uric acid stones in the kidney and urinary system, while increasing urine volume, alkalized urine and reducing the dosage are beneficial for managing this severe complication (FitzGerald et al., 2020; Hui et al., 2017; Yu K. H. et al., 2018). Recently, novel urate transporter 1 (URAT1) inhibitors with higher selectivity, including SHR4640 (Lin et al., 2021), verinurad (Tan et al., 2017), dotinurad (Taniguchi et al., 2019), which have garnered significant attention in the development of anti- hyperuricemia therapy. However, safety concerns have hindered the further development of URAT1 inhibitors, as symbolized by the forced withdrawal of lesinurad.

A large number of literatures have documented that traditional Chinese medicine (TCM) and its active ingredients are potential and potent candidates for the treatment of hyperuricemic nephropathy. Previous studies have found that TCMs effectively attenuate the progression of HN mainly by inhibiting hepatic XOD activity, modulating urate transporters, suppressing inflammatory responses, and ameliorating oxidative stress and renal fibrosis (Yang et al., 2022). With long-term clinical application in humans, the hypouricemia and renoprotective potencies of TCM and TCM formulas have been verified in preclinical and clinical trials, such as Si Miao Pills (Zhang Y. et al., 2023), *Cichorium intybus* L. (Jin et al., 2018), and fisetin (Ren et al., 2021), etc. Considering the potential role of the interaction between the intestine and kidneys in the pathogenesis and treatment of HN, we propose the hypothesis that soluble uric acid and urate crystals could disrupt the physiological function and structure of renal and intestinal systems by regulating multiple intracellular signals, thereby promoting renal fibrosis and furthering the progression of HN. Meanwhile, this paper summarizes and analyzes TCM formulas and single herbs with dual-regulatory effects of the intestine and kidneys in HN treatment in, providing a new perspective and research direction for TCMs.

## 2 The pathogenesis of HN

Growing evidence suggests that both soluble uric acid and MSU crystals can insult renal function and structure through various mechanisms (Mei et al., 2022; Ponticelli et al., 2020; Weaver, 2019). Additionally, the intestine plays a role in the pathogenesis of HN by regulating the metabolism and transport processes of soluble uric acid and MSU crystals (Grelska et al., 2023; Tseng et al., 2020).

### 2.1 The metabolism and transport of purine and uric acid

**FIGURE 1:**
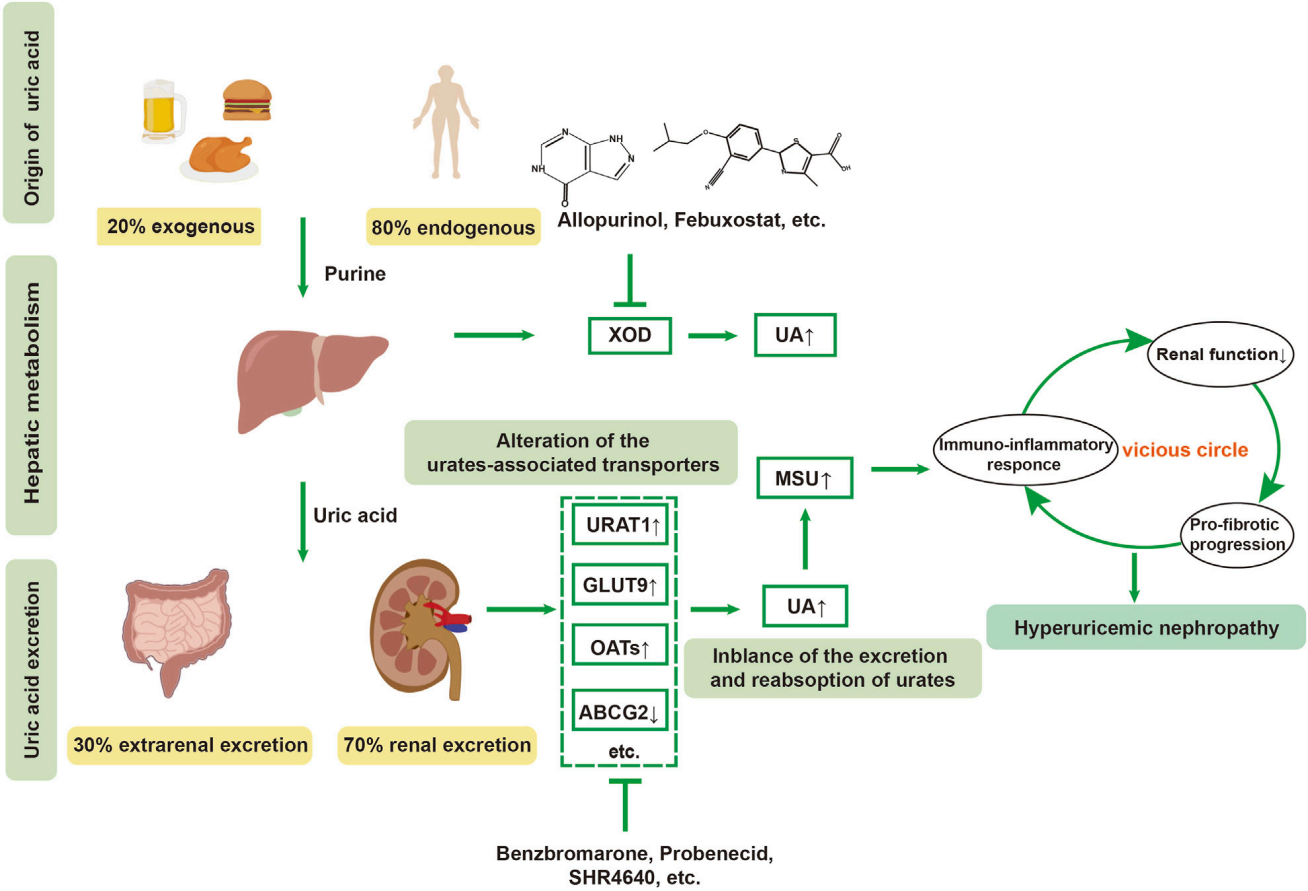
Pathogenesis of hyperuricemic nephropathy (HN). HN is a renal dysfunction secondary to uncontrolled long-term hyperuricemia. Dysfunction of urate-related transporters, such as urate transporter 1(URAT1), glucose transporter 9 (GLUT9), ATP-binding cassette subfamily G member 2 (ABCG2), and organic anion transporters (OATs), lead to the abnormal metabolism of urates in the kidney. The chronic elevation of uric acid (UA) and monosodium urate (MSU) crystals exacerbate the vicious cycle within the kidney through promoting oxidative stress, immune-inflammatory response, renal dysfunction and pro-fibrotic progression, eventually leading to HN.

Uric acid (UA) is the end product of purine metabolism, catalyzed by xanthine oxidase (XOD), which is mainly expressed in the liver. In addition to XOD, blood uric acid levels are dynamically regulated by various transporters in the kidney and gut, as well as by some oxidants or nitric oxide (Gersch et al., 2008; Imaram et al., 2010). In human beings, a physiological level of uric acid (3–5 mg/dL) exists in the bloodstream in the form of soluble urates, while high concentrations of urates that exceed its solubility in the blood (serum uric acid >6.8 mg/dL) contribute to MSU crystalline deposits. Approximately two-thirds of uric acid excretion occurs through glomerular filtration and tubular reabsorption, which relies on urate transporter proteins in the kidneys, including URAT1 (encoded by the *SLC22A12* gene), glucose transporter 9 (GLUT9, encoded by the *SLC2A9* gene), and organic anion transporter 4 (OAT4, encoded by the *SLC22A11* gene). Generally, serum uric acid (sUA) levels are modulated dynamically by two categories of transporters: urate reabsorption-promoting transporters (URAT1, GLUT9, sodium-dependent phosphate transporter protein 1 (NPT1), and NPT4 (Maiuolo et al., 2016), OAT4, OAT10, etc.) and urate excretion-promoting transporters (ABCG2, OAT1, OAT3, etc.). Benzbromarone and probenecid achieve uric acid-lowering function by inhibiting URAT1 and GLUT9, thereby increasing UA excretion (Figure 1). However, some clinical trials reported that 10% hyperuricemic patients treated with benzbromarone could not achieve satisfactory clinical outcomes with a standard therapeutic dose and duration, which might be attributed to its inhibition of OAT1 and ATP-binding cassette subfamily G member 2 (ABCG2). URAT1 facilitates the reabsorption of uric acid from the renal tubules to the tubular epithelial cells and then to the renal interstitium, thus elevating serum uric acid. Conversely, as urate reabsorption-promoting transporters, inhibition of OAT1 and ABCG2 might partially counteract urate-lowering effect of benzbromarone. Therefore, drug discovery targeting urate transporters with high selectivity has attracted significant attention in the pharmaceutical industry, and has been extensively reviewed in elsewhere (Hou et al., 2023; Shi et al., 2022).

Uric acid possesses both antioxidant and pro-oxidant properties (So and Thorens, 2010). It exists in the body in soluble form, as well as crystalline form. An imbalance in uric acid metabolism can result in severe kidney diseases. Research has shown that uric acid metabolism is closely linked to the development of renal disease. Notably, the role of the intestinal tract in the metabolism of purines and uric acid has been confirmed, as about one-third of uric acid is discharged by gut (Eckenstaler and Benndorf, 2021). This is achieved through three main approaches: one involves a serial of urate transporters in the gut, the second one is the direct involvement of the intestinal flora in the metabolic breakdown of purines and uric acid (Table 1), and the third involves metabolites such as short-chain fatty acids (e.g., acetate, butyrate) produced by intestinal bacteria provide energy to intestinal epithelial cells, supporting the excretion of uric acid. Currently, it is well-acknowledged that the uric acid transporters in the intestine include ABCG2, MRP2, MRP4, GLUT9, MCT9, NPT4, its homologs, OAT10, and YgfU (Xu et al., 2016). As a potent anti-hyperuricemic agent, most attention has been concentrated on the development of URAT1 inhibitors, such as dotinurad, epaminurad (URC-102) and XNW3009. In addition, ABP-671 (developed by New Elemental Pharmaceuticals) has emerged as a promising therapeutic candidate due to its concurrent inhibition of URAT1 and GLUT9, demonstrating synergistic dual-target inhibition of urate reabsorption. clinical trials have demonstrated a 30% improvement in urate-lowering efficacy compared to single-target agents, particularly in treatment-refractory populations (Dong et al., 2019). Recently, Granados et al. discovered a transport route from gut microbes to the liver, and then to kidney-mediated OAT1 transport for the gut-derived metabolites (e.g., indolyl sulfate, p-cresol sulfate, deoxycholate, etc.), indicating a central role for kidney urate transporters in modulating gut flora-dependent metabolism (Granados et al., 2023). Mechanistically, OAT1 plays a role in transporting uremic solutes or uremic toxins in the circulation by directly interacting with multiple gut-derived metabolites (Granados et al., 2023). Nevertheless, the specific role of urate transporters in modulating the intestinal microbiome and its metabolites needs further investigation to discover novel therapeutic strategies for delaying the progression of HN.

**TABLE 1:** The intestinal flora involved in hyperuricemia.

Intestinal flora	Species	References
*Bacteroides caccae* and *Bacteroides xylanisolvens*↑; *Faecalibacterium prausnitzii* and *Bifidobacterium pseudocatenulatum*↓	Gout patient	Guo et al. (2016)
*Alipipes, Dialister*, *Roseburia*, *Gemmiger*, and *Faecalibacterium*↑; *Bifidobacterium, Klebsiella*, and *Clostridium*↓	Asymptomatic hyperuricemia patient	Yang et al. (2021)
*Bacteroides*, *Parabacteroides, Gemella*, *Lactococcus*, *Anaerostipes*, *Dorea*, *Anaerotruncus*, *Allobaculum*, *Holdemania*, *Desulfovibrio*, *Morganella*, and *Proteus*↑; *Rothia*, *Collinsella*, *Prevotella*, *Odoribacter*, *Lactobacillus*, *Streptococcus*, *Clostridium*, *Dehalobacterium*, *Ruminococcus,* and *Anaeroplasma*↓	SD rat (10% high-fat feed)	Yu et al. (2018b)
*Bacteroidetes* and *Proteobacteria*↑; *Firmicutes*↓	Mouse (potassium oxazinate)	Cao et al. (2022a)

*Escherichia coli* and *Aspergillus* spp., which secrete XOD have been verified to catalyze purines into uric acid in the intestinal tract, thus reducing intestinal absorption of dietary purines (Crane et al., 2013). Yamada et al. (2016) discovered that *Lactobacillus* spp. regulate serum uric acid levels by uptaking and utilizing purines. The PICRUST analysis indicated that hyperuricaemia was associated with prominent alterations in gut microbiota involved in nucleotide and lipid metabolism. Allopurinol and benzbromarone exhibited uric acid-lowering potency by modulating microbial genera in hyperuricaemic rats, characterized by an increase of *Bifidobacterium* and a decrease of *Adlercreutzia Anaerostipes* (Yu Y. et al., 2018). Surprisingly, these two microbial genera have been confirmed to be closely related with the reduction of UA. Moreover, Spearman analysis showed a strong correlation between the increased in the genus *Adlercreutzia* and the trimethylamine-N-oxide (TMAO) levels in CKD patients with low glomerular filtration rates (GFRs, GFR < 7 mL/min/1.73 m^2^) (Xu et al., 2017), which could further exaggerate hyperuricaemia. Therefore, it can be speculated that intestinal flora-derived metabolites with nephrotoxic properties, such as TMAO, raise blood UA by injuring renal function (Yu Y. et al., 2018). However, beneficial intestinal flora indirectly moderate serum UA levels by producing small molecule metabolites, such as short-chain fatty acids (SCFAs), including butyrate, and propionate (Chu et al., 2021), etc. SCFAs have been proved to play a direct and indirect role in regulating metabolism, inflammation, immune response, and maintenance of mucosal barrier function (Mann et al., 2024). Growing evidence has demonstrated that gut-derived SCFAs, mainly produced by *anaerobic bacilli*, *bifidobacteria*, *fungi*, *streptococci*, and *Lactobacillus*, regulate host health through energy regulation, intestinal mucosal barrier, and immune regulation, which are involved in water-electrolyte homeostasis, oxidative stress and immuno-inflammatory responses in the gut. Previous studies have found that SCFAs could significantly reduce the serum uric acid levels in HUA mice induced by potassium oxonate and hypoxanthine. Moreover, SCFAs supplementation has a dose-dependent inhibitory effect on renal transporters, URAT1 and GLUT9, with the inhibitory effect of SCFAs on GLUT9 being reversible. Activating G protein-coupled receptors (GPCRs, such as GPR41 and GPR43) (Li Y. et al., 2022) and inhibiting the activity of histone deacetylase (HDACs) (Thomas and Denu, 2021), intestinal SCFAs might attenuate the immuno-inflammatory response of gut and other organs induced by hyperuricemia through activating GPCRs and PI3K/AKT/mTOR signaling (Zhao et al., 2018) pathways and weakening HDACs activity. However, the specific mechanism needs further investigation.

### 2.2 The mechanistic role of soluble uric acid (SUA) in the renal dysfunction

SUA has various effects on the kidney, including pathological and physiological processes such as renal arteriopathy, glomerulosclerosis, tubular injury, interstitial fibrosis, and tubular hypertrophy (Jing et al., 2020). Studies have reported that SUA can act as an antioxidant, scavenging unpaired oxygen, oxygen radicals, and peroxynitrite, as well as chelating transition metals. Its antioxidant capacity accounts for half of the total antioxidant capacity of human plasma (Kumar et al., 2015). As demonstrated, physiological concentrations of SUA inhibit the expression of TNF-α and IL-1β-induced inflammatory factors in primary porcine chondrocytes and exert a protective effect on cartilage in collagen-induced arthritis in mice (Lai et al., 2017). Interestingly, SUA exhibits pro-oxidant and pro-inflammatory properties in several studies (So and Thorens, 2010). SUA induces vascular endothelial injury, which is closely related to micro-inflammation, oxidative stress, and lipid metabolism disorders. Notably, the modeling concentration of uric acid used *in vitro* experiments reached 20 mg/dL (namely, 1,200 μmol/L) (Yin et al., 2019), which is much higher than the common pathological levels in hyperuricemic individuals. Therefore, it is suggested that the modeling conditions for hyperuricemia and HN should be consistent with the actual pathological situations.

Once inside the cells, SUA acts as a pro-oxidant molecule, activating fibrotic signaling pathways, including AMPK, ERK1/2, PI3K/Akt, JAK/STAT, NF-κB, and the NLRP3 inflammasome, which trigger oxidative stress and inflammatory responses in the kidney (Li K. et al., 2023). In addition to stimulating an increase in nicotinamide adenine dinucleotide phosphate (NADPH) oxidase activity and reactive oxygen species (ROS) production (Sautin et al., 2007), low concentrations of supraphysiologic SUA act as a pro-oxidant, playing a role in renal pathophysiology. Studies have demonstrated that mild hyperuricemia in mice that underwent 5/6 nephrectomy, exhibited significant renal injury and inflammatory responses (Omizo et al., 2020). In contrast, febuxostat protected the kidneys from hyperuricemia, suggesting the protective role of a uric acid-lowering strategy targeting XOD in the kidneys. Given that hyperuricemic nephropathy is a progressive condition, the pro-oxidant and pro-inflammatory roles of SUA need to be clarified more specifically at different stages of CKD progression.

### 2.3 The mechanistic role of monosodium urate (MSU) crystal in the renal dysfunction

In clinical observations, MSU deposits can be detected by ultrasonography and dual-energy CT examination in the renal tissues and joints that have been affected. As a damage-associated molecular pattern (DAMP), MSU deposition activates a local immuno-inflammatory response, leading to the activation of the NOD-like receptor protein 3 (NLRP3) inflammasome, which produces and releases active IL-1β and IL-18. This alteration initiates the amplification of the inflammatory cascade and profibrotic degeneration in the kidney, ultimately causing glomerular and tubular pathology (Mei et al., 2022). Notably, it has been found that MSU affects the binding of the uric acid transporter URAT1 to Numb, a protein that controls the endocytosis process (Wu et al., 2015), as well as OAT1 membrane endocytosis (Wu et al., 2016). This leads to elevated URAT1 expression and reduced OAT1 expression, which exacerbate the imbalance in urate metabolism, thereby deteriorating renal function. In addition, MSU promotes the apoptosis of kidney cells by inducing the production of reactive oxygen species, increasing nitric oxide synthase activity and mitochondrial caspase enzymes activity, as well as the activation of apoptosis-dependent signaling pathways (Choe et al., 2015), thus exacerbating kidney injury. It cannot be ignored that MSU accumulates in the urinary system, transforming from urate crystals into urate stones, which increases the risk of obstructive nephropathy, urinary tract infections, and kidney damage (Jiang C. et al., 2023). Therefore, it is generally believed that local immuno-inflammatory responses, renal fibrosis, apoptosis, and renal obstruction are responsible for the renal injury caused by MSU crystals.

### 2.4 The imbalance of intestinal immune barrier promotes HN progression

**FIGURE 2:**
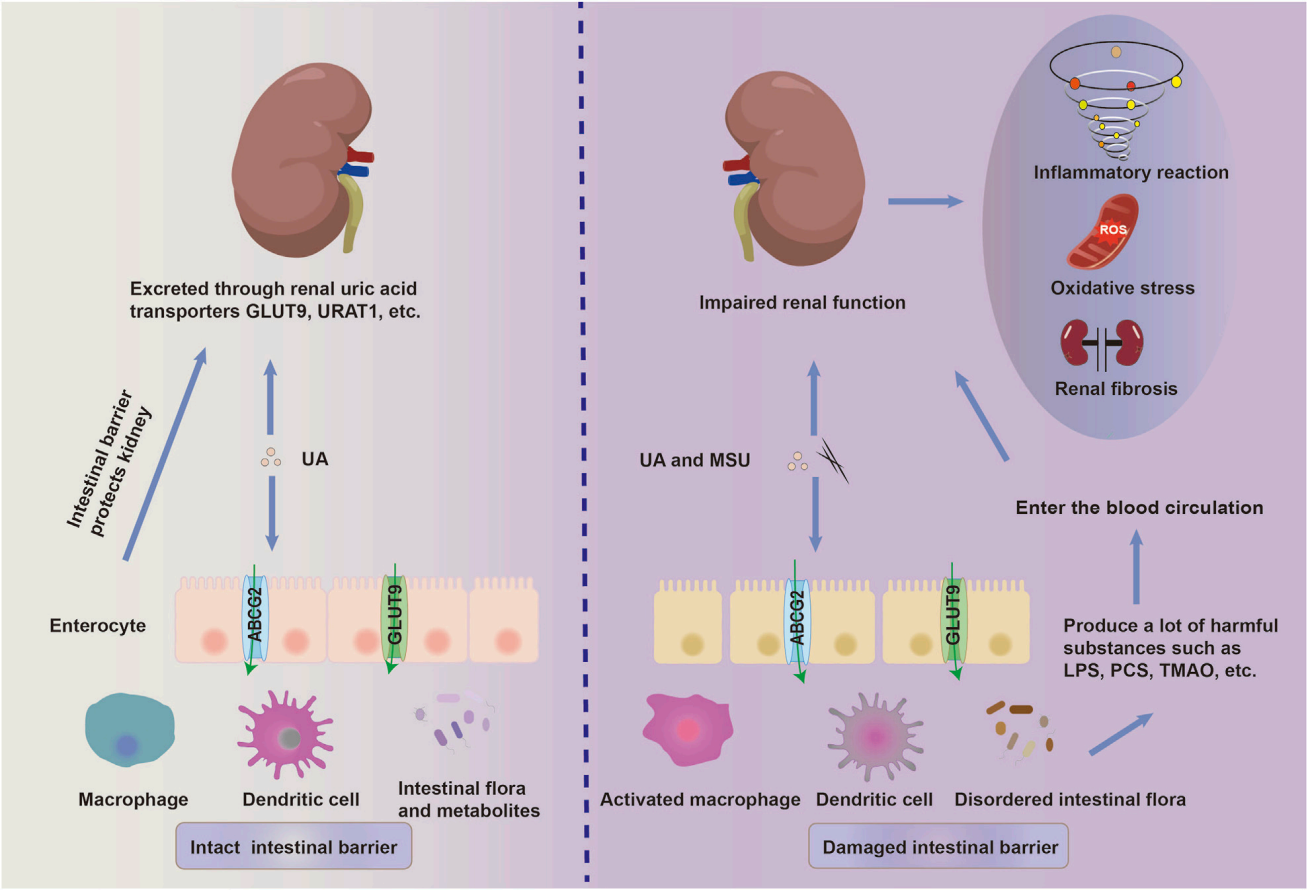
The disruption of the intestinal mucosal immune system promotes the progression of HN. A high-fructose and high-purine diet, along with a poor lifestyle, destroys the intestinal ecology. This disruption promotes the release of lipopolysaccharide (LPS), *p*-cresyl sulfate (PCS), trimethylamine-N-oxide (TMAO), and other toxic substances. Accompanied by chronic hyperuricemia, intestinal pathogenic microorganisms and their toxic substances synergistically contribute to the breakdown of the intestinal immune barrier through the activation of intestinal innate immunity and injury to the intestinal epithelial cells. These gut-derived harmful metabolites and pro-inflammatory mediators enter the kidney via the bloodstream and further aggravate the renal dysfunction by activating inflammation, oxidative stress and fibrosis.

Currently, the pathophysiological role of gut microbiota has been confirmed in various chronic kidney diseases. It has been discovered that patients with gout and hyperuricemia exhibit an imbalance in intestinal ecology, along with increased levels of harmful metabolites in the serum and intestines (Guo et al., 2016). The intrinsic barrier of the intestinal mucosa consists of intestinal epithelial cells, associated connective structures, and robust immunity. Intestinal epithelial cells protect the intestinal barrier by forming physical and biochemical barriers against pathogenic microorganisms, thus participating in the regulation of the mucosal immune system (Yang et al., 2023). When the intestinal barrier function is impaired, endotoxins released by pathogenic microorganisms enter the bloodstream from the intestinal tract, which tends to trigger a series of immune-inflammatory responses (Peterson and Artis, 2014) (Figure 2). Guo et al. (2019) discovered that hyperuricemic mice, in which the uric acid oxidase gene was knock out, showed intestinal barrier dysfunction, subsequent elevated intestinal permeability, and exacerbated renal dysfunction, presumably associated with uric acid-induced inflammatory responses. This suggests a close relationship between hyperuricemic-induced kidney insult and the disruption of intestinal immune barrier. Meanwhile, SUA upregulates the expression of urate transporters PDZK1 (PDZ domain-containing protein 1) and ABCG2 in HT-29 (human colon cancer cells) and Caco-2 cells (human colorectal adenocarcinoma cells) by activating the TLR4-NLRP3 inflammasome and PI3K/Akt signaling pathways, thereby promoting the excretion of uric acid in intestinal cells (Chen et al., 2018). Conversely, previous studies have considered the protective role of SUA in NSAID-induced enteropathy due to the antioxidant effect of uric acid. Yasutake et al. (Yasutake et al., 2017) demonstrated that the oral administration of uric acid and intraperitoneal injection of inosinic acid and potassium oxonate ameliorated NSAID-induced enteropathy in mice by decreasing oxidative stress, lipid peroxidation, and cytotoxicity induced by indomethacin. This implies the potential therapeutic role of SUA in inflammatory enteritis.

In addition to maintaining urate homeostasis by modulating urate transport-related proteins in the intestine, the gut macrobiotic communities and their metabolites affect hyperuricemia and HN progression by regulating “chronic low-grade inflammation” and activating pro-fibrotic signaling. In addition to its pro-inflammatory role in the progression of renal fibrosis, serum urate, together with pro-inflammatory cytokines and mediators, aggravates renal fibrosis via multiple signaling pathways, including PI3K/AKT/mTOR, MAPK/NF-κB-NLRP3 inflammasome, IL-6/JAK2/STAT3, TGF-β/SMAD3, and Wnt/β-catenin signaling pathways. More recently, the roles of pyroptosis, neutrophil extracellular traps formation (Wu et al., 2024), and ferroptosis (Li et al., 2024) in HN have been emphasized, suggesting novel potential approaches for treating progressive HN.

Hyperuricemic animals induced by high-fat and high-fructose diets and alcohol abuse exhibit a disordered gut microbiome, which parallels that seen in hyperuricemic and gouty patients (Zhang C. et al., 2022). The concept of the “gut-kidney axis” was introduced by Meijers et al., in 2011 (Meijers and Evenepoel, 2011), highlights the bidirectional relationship between the gut microbiota and kidney function. Moreover, insoluble fiber from diets and probiotic supplements attenuate renal injury and fibrosis induced by hyperuricemia by restoring the gut microbiota and SCFAs (Li Y. et al., 2022). Furthermore, a growing body of literature has demonstrated that strategies targeting the gut microbiota and inflammation can alleviate renal fibrosis in hyperuricemia (Lv et al., 2020; Pan et al., 2020). However, well-designed experiments are needed to distinguish whether alterations in the gut microbiota are a primary cause or secondary to hyperuricemia. Notably, the role of gut therapy in HN appears to change dynamically with disease progression and the individual differences of the patients. In patients with early-stage HN or those exhibiting compensatory enhancement of intestinal uric acid excretion, therapies targeting the gut microbiota (e.g., probiotics, fecal microbiota transplants) may serve as a core therapeutic strategy. These interventions aim to restore microbial imbalance and alleviate uric acid accumulation. Conversely, in cases of advanced renal impairment, a combined approach integrating renoprotective therapies with gut-targeted interventions may be required to optimize patient management. In summary, the relationship between the gut and the kidney influences the disease process by regulating urate metabolism and transport processes. Therefore, this paper summarizes single herb and TCM formulas that have dual-regulatory effects on both the gut and the kidney. This summary aims to develop new perspectives and research ideas for the clinical treatment of hyperuricemic nephropathy and the development of TCMs drugs.

## 3 The dual-regulatory effect of intestine and kidneys of TCMs for HN treatment

### 3.1 TCM formulas

#### 3.1.1 Regulating intestinal flora and metabolites

Ermiao San, a traditional Chinese medicinal compound, reduces uric acid levels, attenuates glomerular atrophy, and diminishes vacuolar degeneration of renal tubular epithelial cells by inhibiting XOD activity. It has been shown to have preventive and therapeutic effects on hyperuricemia and relate conditions (Shan et al., 2021; Wei et al., 2018). Bining Decoction is a Chinese medicinal preparation derived from Ermiao San, has the efficacy of clearing heat, inducing dampness and eliminating edema. Studies have found that Bining Decoction improves disease-related imbalances *in vivo*, inhibits XOD activity and JAK2 expression within the JAK/STAT pathway, regulates the intestinal flora, and enhances the intestinal barrier. All of these actions play crucial roles in modulating inflammatory factors and apoptotic pathways (Huang et al., 2022).

When administered via enema, the Rhubarb Compound Preparation treats chronic kidney disease by regulating autophagy, apoptosis, intestinal flora and barrier function (Zou et al., 2012; Xiang et al., 2020; Zhang F. et al., 2023). More recently, a systematic review and meta-analysis revealed that rhubarb (*Rhei Radix et Rhizoma*) has a positive therapeutic effect on chronic renal failure (Huang W. et al., 2023). However, as a hospital-prepared formulation, the efficacy and safety of rhubarb-based TCMs therapy needs to be evaluated by high-quality clinical trials.

Compound Tu-Fuling Oral-Liquid (CoTOL), a clinically used Chinese herbal formula for the treatment of hyperuricemia and gout, works by affecting XOD activity and regulating the intestinal flora to reduce uric acid production (Gao et al., 2020). The results of a randomized double-blind controlled trial showed that CoTOL was effective in reducing sUA levels and in preventing acute arthritic flares in gouty patients (Xie et al., 2017). However, a short observation period and the absence of a positive control affect the reliability and validity of this trial. As the main ingredient, *Poria cocos* can significantly improve renal function, glomerular atrophy, and tubular dilation in hyperuricemic rats. Furthermore, its nephroprotective effect is achieved by increasing the abundance of *Bacteroidetes*, *Alistipes* and *Parabacteroides* in the intestinal tract, and decreasing the relative abundance of *Firmicutes*, thereby regulating the intestinal flora (Wang K. et al., 2022).

As a Tibetan medicine, Shiwuwei Rupeng Pills downregulate the expression levels of tumor necrosis factor (TNF), STAT3, and ALB mRNA, while upregulating the expression levels of PPARG mRNA in the renal tissues of rats with HN. These actions inhibit renal inflammatory factors and improve renal injury. Furthermore, the pills play a therapeutic role in treating HN by regulating the structure of the intestinal flora and the expression of signaling pathways and related targets, such as AGE-RAGE, interleukin-17 (IL-17), TNF, and others (Omizo et al., 2020; Xie et al., 2022). Clinical trials shows that after 3–6 courses of treatment, arthritis redness, swelling, and pain can be effectively alleviated, and the therapeutic efficiency can reach 93% (La and Tai, 2018), however, the specific mechanism requires further research.

#### 3.1.2 Regulating intestinal urate-related transporters

Dendrobium officinalis Six Nostrum, which adds *Dendrobium ironpierre* to the basis of Si-Miao Pill, effectively regulates the expression levels of urate transporter proteins URAT1, ABCG2 and PDZK1 in the kidneys, as well as intestinal GLUT9, ABCG2, and CNT2 proteins, thereby reducing the level of uric acid. It also inhibits the LPS/TLR4/NF-κB signaling to reduce the secretion of renal inflammatory factors (Chen et al., 2020). Additionally, Dendrobium officinalis Six Nostrum significantly downregulates CNT2 protein expression, which reduces purine transport from the intestine back to the blood and inhibits XOD activity, leading to a decrease in uric acid production. Furthermore, it restores the protein expression of tight junctions, such as ZO-1 and claudin-1, thereby protecting the intestinal barrier function (Ge et al., 2023).

The combination of *Polyonum Cuspidatum* and *Ramulus Cinnamomi* reduces uric acid reabsorption by inhibiting the overexpression of the renal transporter protein URAT1 at both mRNA and protein levels. It also increases uric acid secretion by promoting the expression of the renal transporter protein OAT3 at the mRNA and protein levels, as well as the small intestinal ABCG2 (Shi et al., 2016; Zhu, 2018).

Miao medicine Tongfengting reduces uric acid levels through several actions: it downregulates the intestinal ABCG2 gene, and downregulates the *URAT1* gene (Cao et al., 2021). Furthermore, it decreases the protein expression associated with renal injury, such as KIM-1, NGAL, TIMP-1, and MCP-1, thereby playing a role in protecting renal function (Cao Y. P. et al., 2022).

#### 3.1.3 Inhibiting intestinal immuno-inflammation

Yiyi Fuzi Baijiang Powder, derived from Zhang Zhongjing’s *The Essentials of the Golden Chamber*, has anti-diarrhea activity. Modern pharmacological studies have found that Yiyi Fuzi Baijiang Powder repairs intestinal mucosal damage, regulates immune balance and intestinal flora, and reduces inflammation and oxidative stress damage (Wang S. N. et al., 2023; Wei et al., 2023). Clinical research has showed that Yiyi Fuzi Baijiang Powder significantly reduces uric acid levels in patients with hyperuricemia in CKD stages 1–3. However, further exploration of the mechanisms affecting renal function is still needed (Zhao, 2023).

#### 3.1.4 Modulation in multiple pathways, aspects and organs

Quzhuo Tongbi Recipe is effective in strengthening the spleen and kidney, and possesses anti-inflammatory and analgesic properties. It was found that Quzhuo Tongbi Recipe regulates the expression of the urate transporter protein ABCG2, regulates the intestinal microbiota and regulates cell differentiation through PI3K-AKT-mTOR pathway. It also downregulates the levels of amino acids dependent on the fermentation of intestinal flora, increases the abundance of intestinal butyrate-producing bacteria and the expression of their metabolites SCFAs, inhibits the production of intestinal inflammatory factors, and restores the function of the intestinal barrier (Song et al., 2023; Wen et al., 2021; Liu et al., 2019).

As a classic formula, Si Miao San is mainly composed of *Atractylodes Lance* (Thunb.) DC, *Phellodendri Chinensis* Cortex*, Achyranthes bidentata, Coix lacryma-jobi* var. It has the efficacy of clearing heat and dispelling dampness. The treatment of HN with Si Miao San is mainly reflected in its ability to inhibit uric acid production and promote uric acid excretion. This is achieved by regulating the transduction of JAK2/STAT3 and PI3K/Akt signaling pathways, increasing the expression of the anti-inflammatory factor IL-10, and decreasing the expression of pro-inflammatory factors such as TNF-α, IL-6, and IL-1β. Additionally, it regulates the uric acid transporter proteins URAT1, GLUT9, and OAT1 (Cao L. et al., 2022; Zhang Y. et al., 2023). Further studies have found that Si Miao San inhibits NLRP3 inflammasome to reduce intestinal inflammation and alters the composition and function of the intestinal flora (Lin et al., 2020; Zhang, et al., 2022e). Clinically, a derivative of Si Miao San is mainly applied, with Tu Fu Ling and *Lonicera japonica* added to improve renal function (Zhang G. J. et al., 2022). Systematic evaluations and meta-analysis have shown that Modified Si Miao Decoction exerts therapeutic effects on gout through its anti-inflammatory and uric acid-lowering effects (Liu et al., 2017). However, due to poor quality of research methodology adopted in the included clinical trials, there is a strong need for multi-center randomized trial designs with sufficient samples to further validate its efficacy and safety.

The Bi Xie Fen Qing Yin improved renal inflammation, fibrosis and water-fluid metabolism disorders in mice by inhibiting the activation of the NLRP3 inflammasome and pro-fibrotic signaling. It also had a certain modulating effect on intestinal flora disorders, metabolite abnormalities, intestinal inflammation, intestinal barrier damage, and uric acid excretion in mice (Lin, 2022). In clinical practice, the combination of Bi Xie Fen Qing Yin with febuxostat was found to have more significant efficacy in improving renal function in patients with HN after drug interventions were administered (Li X. Q. et al., 2022), However, the trail conducted in a small sample size limits the clinical application.

The *Cichorium intybus* L. Formula inhibited the expression of STAT3, vascular endothelial growth factor A (VEGFA) and SIRT1. It also regulated the diversity and community structure of intestinal flora and improved the symptoms of HN (Amatjan et al., 2023). In addition, chicory was found to regulate the expression of the intestinal ABCG2 protein to promote uric acid excretion (Wang et al., 2017).

Sishen Pill reduced the level of TMAO in serum, kidney and small bowel by regulating *Firmicutes*, *Succinum hispidum* and *Clostridium butyricum.* It also prevented the transmission of inflammatory factors, such as NLRP3 and IL-1β, through the “gut-kidney axis,” thereby improving intestinal barrier injury and renal fibrosis (Xie et al., 2024). (Table 2)

**TABLE 2:** The effects of TCM formulas on kidney and intestine.

TCM formula	Ingredients	Model	Kidney effects	Intestinal effects	References
Bining decoction	*Dioscorea opposita* Thunb, *Plantago ovata* Forssk, *Atractylodes macrocephala* Koidz, *Coix lacryma-jobi* L., *Eucommia ulmoides* Oliv, *Cyperus rotundus* L., *Ligusticum chuanxiong* Hort, *Ligusticum wallichii* Franch, *Lonicera japonica* Thunb	HN mice (adenine and yeast)	UA↓, BUN↓, CREA↓, renal body mass index (RBMI)↓	There was a significant change in the abundance of *E. faecalis*, *E. romobacterium*, *E. bifidum*, *E. anisopliae*, *E. odorata*, *E. furfur NK4A136 group*, *unclassified E. furfur*, *E. rossbarella*, *E. furfur*, *Lactobacillus* spp., *E. dublinii*, *S. rhamnosus*, and *B. tularensis* in the comparison of abundance between the groups	Huang et al. (2022)
Rhubarb compound	*Rheum rhabarbarum* L., *Taraxacum officinale* F.H. Wigg, *Moringa oleifera* Lam., etc.	Patients with chronic renal failure in the nondialysis phase	BUN↓, CREA↓, UA↓	*Bifidobacteria* and *Lactobacillus*↑	Zou et al. (2012)
Compound Tu-fuling oral-liquid	*Poria cocos* (Schw.) Wolf, *Dioscorea villosa* L., *Curcuma longa* L., *Sambucus nigra* L., *Datura stramonium* L., *Zea mays* L., *Coix lacryma-jobi* L.	HUA rats (potassium oxide)	UA↓	*Akkermansia, Bacteroides* and *Beppe’s algae*↑, *Beppe’s algae*↓	Gao et al. (2020)
Shiwuwei rupeng pills	Fifteen flavors, including frankincense (*Boswellia carterii* (Birdw.) Birdw. ex Baker), *Cassia tora*, *Galium davidii*, *Sapindus mukorossi Gaertn*, iron rod hammer (made), etc.	HN rats (adenine and ethambutol)	UA↓, CREA↓, BUN↓	*Acteroides*, *Akkermansia*, *Ralstonia* and *Prevotellaceae Ga6A1 group genera*↓, *Lactobacillus* and *Ruminococcaceae UCG-014*↑	Xie et al. (2022)
Dendrobium officinalis six nostrum	*Dendrobium officinale*, *Rhizoma Atractylodis Atractylodes macrocephala* Koidz, *Rhizoma Atractylodis Macrocephalae*, *Coix lacryma-jobi* L.	HUA rats (hypoxanthine and potassium oxonate)	sUA↓, FUA↓	Modulation of intestinal uric acid transporter protein GLUT9 and ABCG2 protein levels; restoration of ZO-1 and claudin-1 expression to protect intestinal barrier function	Chen et al. (2020), Ge et al. (2023)
Combination of *Polyonum Cuspidatum* and *Ramulus Cinnamomi*	*Atractylodes lancea* (Thunb.) DC. and *Cinnamomum verum* J.Presl	HUA rats (hypoxanthine and potassium oxonate)	sUA↓, CREA, BUN↓, UUA↓	Intestinal uric acid transporter protein ABCG2 expression↑	Shi et al. (2016), Zhu (2018)
Miao medicine Tongfengting	Gypsum Caesarum*, Anemarrhena asphodeloides* Bunge*, Cibotium barometz* (L.) J. Sm, *Lobelia chinensis* Lour, *Coix lacryma-jobi* L., *Tian Qi Cao*, *Phellodendron amurense* Rupr, *stolonifera* Curt, etc.	HUA rats (adenine and potassium oxonate)	sUA↓, CREA↓, BUN↓	Intestinal ABCG2 gene expression↑	Feng et al. (2021)
Yiyi fuzi baijiang powder	*Coix lacryma-jobi* L., *Epiphyllum oxypetalum*, and septoria	Patients with gouty arthritis	UA↓	*Mycobacterium anisopliae and Lactobacillus*↑, *Mycobacterium thickum* and *Mycobacterium anisopliae*↓; IL-6 and CASP3↓; IL-10, PCNA, EGF, Occludin, and ZO-1↑	Wang et al. (2023b), Zhao (2023)
Quzhuo Tongbi recipe	*Poria cocos* (Schw.) Wolf*, Dioscorea opposita* Thunb*,* Cornu Cervi, *Coix lacryma-jobi* L., *Pseudostellaria heterophylla, Morus alba* L., *Curcuma zedoaria* (Christm.) Roscoe, *Ligusticum chuanxiong* Hort, *Psoralea corylifolia* L.	HUA Rats (yeast paste and potassium oxybate)	UA↓	*Collinsella Proteus*↑; *Gemella*, *Anaerostipes* and *Desulfovibrio*↓	Liu et al. (2019)
Quzhuo Tongbi recipe		Gouty arthritis mice (yeast and MSU)	sUA↓	*Mycobacterium anisopliae phylum*; *Coccidioides butyric acidophilus*↑; affected the levels of fecal SCFAs; uric acid transporter protein ABCG2↑; intestinal NLRP3, IL-1β, and TNF-α mRNA levels↓	Wen et al. (2021)
Quzhuo Tongbi recipe		Uox-KO (uric acid oxidase knockout) hyperuricemic and gouty mice	sUA↓, sCREA↓, UUA↓, UCREA↓, UTP↓, UALB↓	*Mycobacterium anisopliae* and *Mycobacterium thickum*↓; *Heterobacterium spp*. and *Candida* spp.↑	Song et al. (2023)
Si Miao San and Si Miao derivative formula	*Phellodendron chinense* Schneid*, Atractylodes macrocephala* Koidz*, Hyssopus officinalis* L., *Coix lacryma-jobi* L., *Poria cocos* (Schw.) Wolf*, Lonicera japonica* Thunb	HUA mice (potassium oxalate and hypoxanthine)	UA↓, BUN↓, CREA↓, UACR↓	*Rumococcus* spp. *_UCG-014, Enterococcus* spp. *Lactobacillus* spp↓; *Allobaculum, Duchenne, Rumococcus* spp. *_NK4A214*, and *Rhodobacter* spp↑. intestinal ABCG2 expression↑	Zhang et al. (2022b)
Bi Xie Fen Qing Yin	*Dioscorea opposita* Thunb, *Eucalyptus*, *Calamus rotang* L., and *Arctostaphylos uva-ursi* (L.) Spreng	HUA mice (adenine and potassium oxonate)	sUA↓, BUN↓, CREA↓, RBMI↓	Zo-1, Claudin-1, and Mucin-3↑; MMP-9↓; Intestinal ABCG2 expression↑; *Bifidobacterium*, *Desulfovibrio*, *Enterobacter*, *Faecalibaculum*, *the Helicobacter*, *Lactobaacilus*, and *Parabacteroides*↑; *uminococcaceae UCG 013* and *Streptococcus*↓	Lin (2022)
*Cichorium intybus* L. formula	*Ch* *Cichorium intybus* L., *Gardenia jasminoides* J.Ellis*, Pueraria mirifica* Craib, *Lilium candidum* L., *Morus alba* L., *Angelica dahurica* (Fisch. ex Hoffm.) Benth. et Hook.f., *araxacum officinale* F.H. Wigg	HN rats (adenine and ethambutol)	UA↓, UREA↓, CREA↑; STAT3, VEGFA and SIRT1↓	*Mycobacterium anisopliae*↓; *Lactobacillaceae*, *Erythrobacteriaceae*, *Lacertidae*, *Ruminococcaceae*, and *Bifidobacteria*↑; intestinal ABCG2 protein expression↑	Amatjan et al. (2023)
Sishen Pills	*Myristica fragrans* Houtt*, Schisandra chinensis* (Turcz.) Baill, *Evodia rutaecarpa* (Juss.) Benth, *Zingiber officinale* Roscoe, and *Ziziphus jujuba* Mill	Diarrhea with kidney-yang deficiency syndrome mouse (adenine and *Folium sennae* decoction)	IL-1β, IL-4, IL-9, and IL-17A↓	*Akkermansia, unclassified Muribaculaceae, and Lactiplatibacillus*↑; *Firmicutes*, *Succinatimonas hippei*, and *Clostridium tyrobutyricum*↓	Xie et al. (2024)

### 3.2 Single herbals

#### 3.2.1 Regulating intestinal flora and metabolites

*Poria cocos*, the dried mycelium of *P. cocos* (Schw.) Wolf from the family Polyporaceae, is known for its dual role as both a medicine and a food. Studies have shown that it improves renal function in hyperuricemic rats by upregulating the expression of the ABCG2 protein (Liang et al., 2021). Additionally, *P. cocos* regulates the beneficial intestinal flora, alleviates renal tubular dilation, glomerular atrophy, and cellular vacuolization in the kidney (Wang K. et al., 2022). The polysaccharide found in *P. cocos* enhances the intestinal barrier in mice and maintains the balance of intestinal flora, contributing to its therapeutic effects (Duan et al., 2023).

Coicis Semen is the dried mature seed kernel of *C. lacryma-jobi L.var.ma-yuen* (Roman.) Stapf, from the family Gramineae. The oil derived from Coicis Semen reduces the imbalance of intestinal flora and inhibits the expression of factors that cause intestinal inflammation. This was achieved by decreasing the levels of *enterococci* and *Enterobacteriaceae*, and increasing the relative abundance of *Bifidobacteria* and *Lactobacillus* in mice (Wang T. et al., 2020). Additionally, an extract from *C. lacryma* has been shown to inhibit XOD activity, thereby reducing uric acid levels and ameliorating renal damage in cases of HN (Sui et al., 2023).

Turmeric, the dried rhizome of *Curcuma Longa L*. from the family *Zingiberaceae*, contains the active ingredient curcumin. Curcumin regulates the structure of the intestinal flora, thereby enhancing the intestinal barrier. It also inhibits XOD activity, alleviates renal tubular interstitial fibrosis, and reduces the production of inflammatory factors and the secretion of PO-1. Additionally, curcumin prevents the activation of the NLRP3 inflammasome signaling pathway. These actions contribute to curcumin’s therapeutic role in treating HN and reducing inflammation (Chen et al., 2019; Shen et al., 2017; Xu et al., 2021).

Astragalus membranaceus, the dried root of *Astragalus membranaceus* (Fisch.) Bge. var. *mongholicus* (Bge.) Hsiao or *A. membranaceus* (Fisch.) Bge., belongs to the legume family. It possesses anti-inflammatory, antioxidant, and immunomodulatory functions (Auyeung et al., 2016). Studies have shown that *A. membranaceus* reduces uric acid levels by regulating intestinal flora and metabolites (Zhao et al., 2022). Furthermore, when fermented with *Bacillus subtilis*, *A. membranaceus* downregulates the expression of uric acid transporter proteins, URAT1 and GLUT9, and inhibits XOD activity, thereby reducing uric acid levels (Wang R. et al., 2023).

Mountain waxberry, the leaf of *Chimonanthus nitens* Oliv. in the genus Waxberry and family *Waxaceae*, restores intestinal homeostasis by regulating the abundance of beneficial intestinal bacteria and the expression of proteins related to the intestinal barrier. Additionally, mountain waxberry regulates XOD activity, influences genes and proteins involved in the reabsorption and secretion of uric acid, inhibits uric acid production, and promotes its excretion, thereby reducing the UA-mediated kidney damage (Meng et al., 2023).

*Andrographis paniculata* is the dried aerial part of *A. paniculata* (Burm. f.) Nees, family Dioscoreaceae. The total saponins of *A. paniculata* reduce uric acid and exert anti-inflammatory effects by regulating intestinal flora and metabolites (Yu et al., 2023). They also inhibit the activities of XOD and adenosine deaminase (ADA), subsequently downregulating the expression of the OAT1, which helps in the urate metabolism.

#### 3.2.2 Regulating intestinal urate transporters

*Stevia*, a member of the Asteraceae family, is rich in flavonoids and chlorogenic acid compounds. It possesses antioxidant, anti-inflammatory, and anti-fibrotic effects. Studies have shown that stevia residue extract improves kidney function in hyperuricemic mice by upregulating the expression of the ABCG2 protein and downregulating the expression of the GLUT9 protein (Mehmood et al., 2019). This was achieved through the MMP-7 and MMP-9/β-catenin signaling pathways, which helped to reduce fibrosis and epithelial-mesenchymal transition (EMT). Additionally, stevia inhibited the activation of the NF-κB/NLRP3 pathway via the AMPK/SIRT1 pathway and modulated the JAK2-STAT3 and Nrf2 signaling pathways, which contributed to the reduction of renal inflammation (Mehmood et al., 2022).

#### 3.2.3 Modulation in multiple pathways, aspects and organs

A water-soluble polysaccharide in *L. japonica* was able to attenuate oxidative stress *in vivo*, reduce kidney injury caused by excess reactive oxygen species and regulate intestinal flora, and downregulate IL-1β, IL-6, TNF-α,COX-2-related inflammatory factors (Sun et al., 2023). It also reduced uric acid levels (Yang et al., 2019). Chlorogenic acid in *L. japonica* extract reduced serum lipopolysaccharide (LPS) levels and downregulated the mRNA expression of IL-18, TNF-α, NLRP3, and Caspase-1. Additionally, it inhibited the activation of TLR4/MyD88/NF-κB signaling pathway in the kidneys, thereby alleviating renal inflammation in hyperuricemic mice. Furthermore, it regulated the intestinal flora, metabolites, and inflammation factors gene expression to ameliorate the inflammatory response (Zhou et al., 2021).

Saffron, the dried stigma of *Crocus sativus* L. from the family Iridaceae, contains the main constituent crocin, a type of flavonoid. Crocin inhibited and reduced the activity of XOD, regulated the expression of renal and intestinal URAT1, GLUT9, and ABCG2 genes and proteins, lowered uric acid levels, and modulated intestinal flora. These actions effectively alleviated the symptoms of HN (Chen et al., 2022).

*Poria cocos* is the dried sclerotium of a fungus, not a plant, and it contains quercetin, which interacts with XOD to reduce uric acid levels (Li X. et al., 2022). It also contains flavonoids that inhibit the expression of oxidative stress and inflammatory-related factors in the kidney, and regulate the expression of genes ABCG2, OAT1, organic cation transporter 2 (OCT2), and organic cation transporters novel 2 (OCTN2) to reduce uric acid levels (Wang et al., 2019). Additionally, *P. cocos* regulates the content of intestinal SCFAs, thereby affecting the intestinal barrier function (Zhao et al., 2021). The contained Astilbin can increase the content of SCFAs and regulate the intestinal flora and the expression of intestinal uric acid transporter proteins, thereby modulating kidney injury and uric acid excretion (Guo, 2020).

Chicory, a traditional Uyghur medicine, comes from the dried above-ground parts or roots of two species: *Cichorium glandulosum* Boiss. et Huet and *Cichorium intybus* L., both belonging to the Asteraceae family. It is used to inhibit uric acid production by regulating OAT2 expression and inhibiting XOD activity. Additionally, it regulates the expression of intestinal flora and intestinal ABCG2, further inhibiting uric acid production and promoting its excretion (Wang et al., 2017). Further studies have found that chicory extract has a comprehensive regulatory effect on the intestinal barrier (Wang et al., 2018). It inhibits the oxidative stress-mediated tissue injury by suppressing the expression of intestinal inflammatory factors. It also reduces the serum levels of D-lactic acid and beta-microglobulin, thereby improving oxidative stress-induced tissue inflammation (Wang et al., 2020b; Wang et al., 2020c).

Rhubarb consists of the dried roots and rhizomes of several species, including *Polygonum palmatum, Rheum palmatum* L., *Rheum tanguticum* Maxim. ex Balf., and medicinal rhubarb *Rheum officinale* Baill. The active ingredient, rhodopsin, indirectly reduces uric acid levels by regulating intestinal *Lactobacillus* (Wu et al., 2020). Rhein directly reduces uric acid levels by downregulating the expression of intestinal GLUT9 (Zhou, 2017). Additionally, it inhibits XOD activity and decreases the expression of inflammatory factors such as IL-1β, TNF-α, PGE2, and TGF-β, which exerts uric acid-lowering and anti-inflammatory effects (Meng et al., 2015). Stewed Rhubarb, as a processed product of rhubarb, has been shown to exert anti-inflammatory and antifibrotic effects by inhibiting the activation of renal NLRP3 and decreasing the mRNA levels of *Il-1β*, *TNF-α*, *Col1a1*, and *Fibronectin* in adenine-induced chronic renal failure (CRF) mice. On the other hand, Stewed Rhubarb increased the abundance of *Bacteroidales* but reducing the contents of *Rikenellaceae* and *Erysipelotrichaceae* in CRF mice, exerting a role in restore microbial balance and intestinal barrier integrity (Wang R. et al., 2022).

*Phellodendron chinense* Schneid. exerted anti-inflammatory effect by inhibiting renal PI3K/AKT, TNF, MAPK, TLR and NLRP3 pathway signaling pathways (Xu et al., 2022). Palmatine alkaloids inhibited XOD activity, downregulated GLUT9 and URAT1 protein expression, and upregulated OAT1 and ABCG2 expression to reduce serum uric acid levels. It also alleviated renal inflammation and oxidative stress caused by hyperuricemia, which contribute to targeting the Keap1-Nrf2/NLRP3 inflammasome signaling pathway (Ai et al., 2023). Furthermore, recent research discovered that phellodendrine, the characteristic component of *P. chinense* Schneid., regulates the composition of intestinal flora and the AMPK/mTOR-mediated autophagy pathway to restore the intestinal mucosa injury induced by intestinal inflammation (Su et al., 2021).

In conclusion, TCM preparations and single herbals alleviate HN symptoms by regulating the urate-related transporters, intestinal flora and metabolites, and immune-inflammatory responses. The involved signaling pathways include MAPK, AMPK/SIRT1, JAK2/STAT3, PPAR/NF-κB, PI3K/AKT/mTOR, MMP9/β-catenin and NLRP3 inflammasome, etc. Besides promoting uric acid excretion by regulating urate transporters, the restorative effects of TCMs on the intestinal and renal dysfunction are attributed to the modulation of multiple targets, pathways and aspects, which are responsible for the anti-HN potential of TCMs (Table 3).

**TABLE 3:** The effects of single herbals on kidney and intestine.

Single herbal	Model	Kidney effects	Intestinal effects	References
Fuling (*Poria cocos* (Schw.) Wolf.)	HUA rats (adenine and potassium oxonate)	UA↓, BUN↓, CREA↓; RBMI↓; ABCG2, OAT1, OAT3 and OCT2 expression↑	*Bacteroides*, *Alistipes*, *Parabacteroides*, *Oscillibacter* and *Roseburia*↑; *Romboutsia, Turicibacter*, *Blautia* and *unidentified_Corynebacteriaceae*↓	Duan et al. (2023), Wang et al. (2022a)
Yiyiren (*Coix lacryma-jobi* L.)	HUA mice (hypoxanthine and potassium oxonate)	UA↓, CREA↓	*enterococci* and *enterobacteria*↓; *bifidobacteria* and *lactobacilli*↑	Sui et al. (2023), Wang et al. (2020a)
Jianghuang (*Curcuma longa* L.)	HN rats (adenine and potassium oxonate)	UA↓, CREA↓, BUN↓, NLRP3 inflammatory vesicles↓	Regulation of *Prevotellaceae*, *Bacteroidaceae*, and *Rikenellaceae*; SCFAs producing bacteria such as *Lactobacillus* and *Lactococcus lactis*↑	Chen et al. (2019), Xu et al. (2021)
Huangqi (*Astragalus membranaceus* (Fisch.) Bunge.)	HN in rats (yeast and adenine)	sUA↓, CREA↓, BUN↓; URAT1, GLUT9 expression↓	Urease-associated genera such as *Fungus spp*, *Bacteroidetes spp, Ruminalococcus spp*, and *Clostridium* spp. *pathogenic genera*↓; SCFA-producing genera↑	Zhang et al. (2022d)
Shanlamei (*Chimonanthus nitens* Oliv.)	HUA mice (potassium oxonate)	UA↓, CREA↓, BUN↓	ZO-1,MUC2 and MUC4 mRNA levels↑; TLR4 and MyD88 mRNA levels↓; *Lactobacillus, Alistipes, revotellaceae_UCG-001* and *Parasutterella abundance*↑	Meng et al. (2023)
Chuanshanlong (*Dioscorea nipponica* Makino)	HUA rats (yeast and potassium oxonate)	UA↓, BUN↓	The phylum Thicket, *Lactobacillus* spp., *Clostridium* spp.*, Rumex* spp., *Lactobacillus rhamnosus* spp., *Lactobacillus coelicolor spp*. and *Lactobacillus royalei spp*.↑; the SCFA content↑	Yu et al. (2023)
Tianyeju (*Stevia rebaudiana* (Bertoni) Bertoni)	HUA mice (fructose and yeast)	UA↓, CREA↓, BUN↓; IL-18, IL-6, IL-1β and TNF-α↓	ABCG protein↑; GLUT9 protein expression↓	Mehmood et al. (2020)
Rendong (*Lonicera japonica* Thunb.)	HUA rats (hypoxanthine and potassium oxonate) and gouty arthritis (MSU)	UA↓, IL-1β, IL-6, TNF-α, COX-2 expression↓	*Thick-walled bacilli/anabolic bacilli*↑; *Lactobacillaceae and Bifidobacteriaceae*↑; intestinal IL-1β and IL-6 mRNA expression↓; ZO-1 and occludin mRNA expression↑	Chien et al. (2009), Zhou et al. (2021)
Xihonghua (*Crocus sativus* L.)	HUA rats (potassium oxonate)	UA↓, CREA↓, BUN↓	*Alloprevotella, Clostridioides, Erysipelatoclostridium, Holdemania (genus)* and *Parabacteroides_goldsteinii*↓; *Roseburia (genus) and Clostridium_*sp. *(Species)*↑; intestinal GLUT9 expression↓ and ABCG2 expression↑	Chen et al. (2022)
Tufuling (*Smilax glabra L.*)	HN rats (yeast and adenine)	ABCG2, OAT1, OCT2 and OCTN2 expression↑	The *thick-walled* and *anaphylactic phyla*↓; *anaphylactic phyla*↑; intestinal ABCG2 expression↑	Wang et al. (2019), Zhao et al., 2021, Guo (2020)
Juju (*Cichorium intybus* L.)	HUA rats (fructose)	sUA↓, UUA↓, FUA↓, CUUA↓, SOD↑, TNF -α↓, IL-6↓; OAT3↑	*E. coli, Enterococcus faecalis*↓; *Bifidobacterium bifidum*↑; intestinal ABCG2 expression↑	Wang et al. (2020b), Wang et al. (2018)
Dahuang (*Rheum palmatum* L.)	HN mice (adenine and ethambutol)	UUR, SCR, UCR, BUR↓; IL-1β, TNF-α, PGE2, TGF-β↓	*Lactobacillus levels* and number of SCFAs↑; GLUT9 expression↓	Ji et al. (2022), Wu et al. (2020)
Huangbo (*Phellodendron amurense* Rupr.)	HUA mice (potassium oxonate and hypoxanthine)	sUA↓, CREA↓, BUN↓	Restore the *lactic acid bacteria* count	Su et al. (2021)

## 4 Challenges and perspectives

**FIGURE 3:**
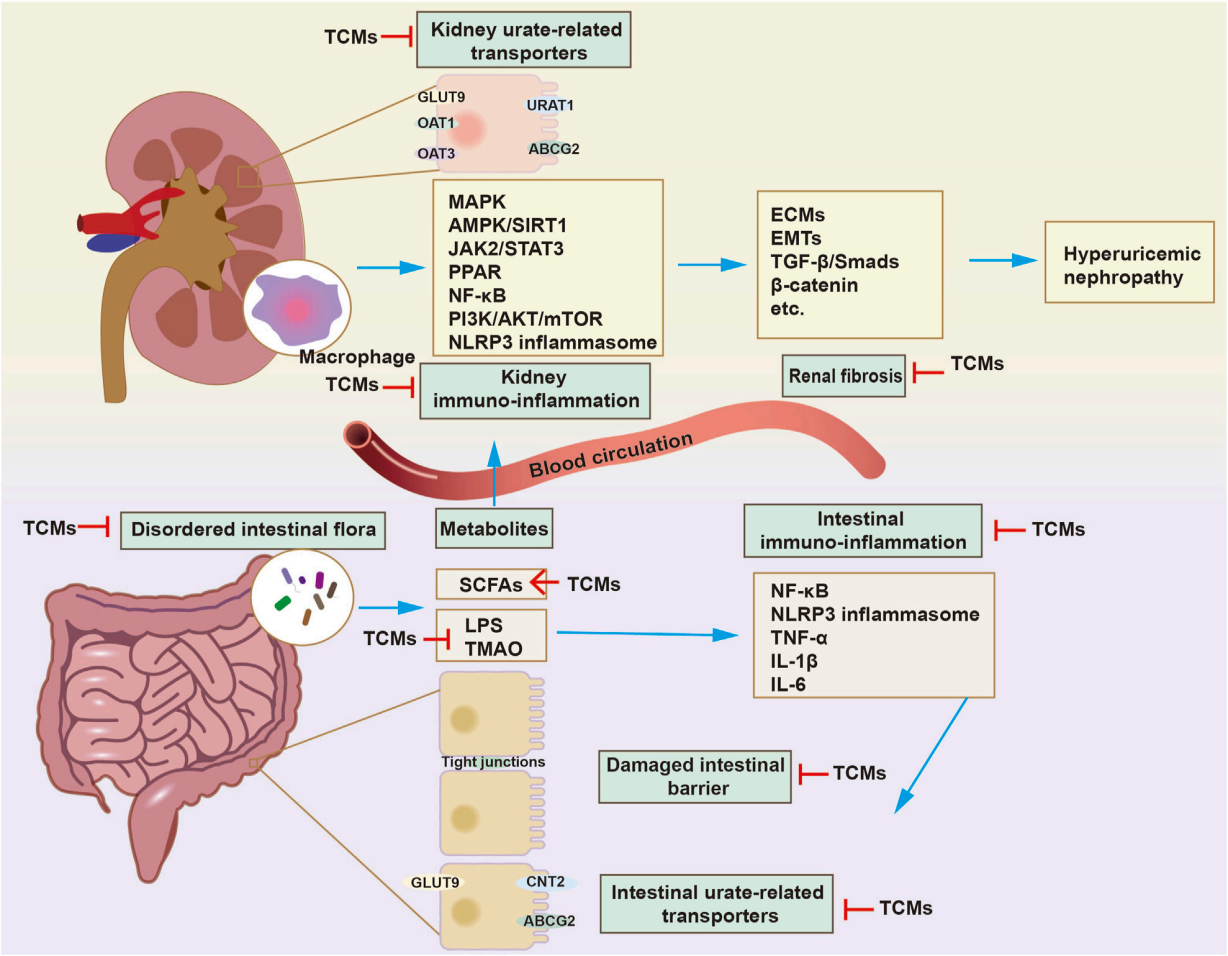
The mechanism of traditional Chinese medicines (TCMs) in delaying HN progression through dual regulation of the gut and kidneys. The treatment of HN with TCMs is mainly achieved by modulating urate-related transporters, oxidative stress, immune-inflammatory reactions, and pro-fibrosis signaling in kidneys. The modulatory effects of TCMs on disordered intestinal flora and metabolites have been discovered. Besides regulating intestinal urate-related transporters, such as concentrative nucleoside transporter 2 (CNT2), ABCG2, GLUT9, TCMs exert a uric acid-lowering effect by increasing the abundance of specific microorganisms that break down purines and uric acid. On the other hand, TCMs restore intestinal immune barrier function by inhibiting the immune-inflammatory response and increasing the expression of tight junctions, thereby inhibiting the entry of enterogenous harmful substances into the kidneys. In general, TCMs delay HN progression through a dual-regulatory effect on the intestines and kidneys.

In recent years, research on the potential role of altered intestine function in hyperuricemia and HN has surged. Thus, this paper systematically reviews the pathogenesis of HN, the effects of SUA and MSU on renal dysfunction, and the role of the intestinal tract in the progression of HN. This article primarily focuses on the advancements in traditional Chinese medicine and single-flavored Chinese medicine for HN management, summarizing and analyzing 13 types of TCM and 13 single herbs. In general, we propose the perspective that traditional Chinese medicines delay HN progression through a dual-regulatory effect on the intestines and kidneys, particularly by the modulation of intestinal flora and metabolites, uric acid transporters and the immune-inflammatory response (Figure 3).

### 4.1 Is intestinal therapy a core mechanism or an adjunctive therapy?

The gut (about 30%) and the kidney (about 70%) are the main pathways for uric acid excretion. When renal function is impaired, intestinal excretion may be compensatorily increased and become a key pathway for lowering serum uric acid. On the one hand, the accumulation of toxins in the circulation induced by the renal dysfunction, leads to an imbalance in the intestinal flora. On the other hand, the alteration of intestinal flora leads to the release of a large number of toxic metabolites and endotoxin, activating the immune-inflammatory response and destroying the intestinal mucosal barrier. This, in turn, aggravates to the renal dysfunction in the hyperuricemia. Therefore, the multiple modulatory effects of TCMs on intestinal excretory capacity (e.g., inhibition of the intestinal urate transporter), the abundance of specific bacteria associated with purine and uric acid metabolism, and the disturbed intestinal flora composition may become an alternative therapeutic strategy for HN.

TCMs play a key role in increasing the beneficial intestinal flora, such as *E. faecalis spp*. and *Bifidobacterium spp*. etc., which aid in digestion and absorption, and stimulate the body’s immune function to protect health. It also regulates the neutral flora, such as *L.* spp., *E. coli, Streptococcus* spp. etc., which, if imbalanced, can lead to an increase in harmful substances and toxins, promoting aging. Additionally, it helps decrease harmful flora, such as *A.* spp. and *Bacteriodesmus* spp. etc., which can reduce intestinal infections (Li X. et al., 2021). Therefore, an imbalance in the intestinal flora may lead to abnormal purine metabolism and increased uric acid production, while microbial metabolites (such as SCFAs) may affect renal inflammation and fibrosis through the “gut-kidney axis.” Intervention of intestinal flora may reduce the source of uric acid and alleviate renal injury. Notably, for patients with significant gut microbiota dysbiosis (e.g., those with chronic inflammatory bowel disease or metabolic syndrome), the gut-kidney axis could be discussed as a central mechanism. In cases of advanced renal impairment, a combined approach integrating renoprotective therapies with gut-targeted interventions may be required to optimize patient management. Growing evidence has revealed that TCMs exhibit anti-HN potential via multi-target, multi-pathway, and multi-approach. Some animal experiments have shown that TCMs (e.g., Bining Decoction, Poria, Astragalus, etc.) can both inhibit uric acid production and restore the balance of intestinal flora, suggesting the centrality of intestinal therapy. However, the causal relationship between intestinal flora and HN is still unknown. Thus, it is suggested that the causative role of gut microorganism in the development and progression of HN should be investigated through the depletion of intestinal flora. Previously, Emal et al. (2017) depleted the intestinal microflora by applying antibiotics, treated with oral administration of faecal suspensions from healthy mice for 3 days, and subsequently established a mouse model of renal ischemia-reperfusion, and found that the intestinal flora exacerbated renal ischemia-reperfusion injury by modulating macrophage maturation and chemotaxis, while faecal transplantation attenuated the renal injury, suggesting that intestinal flora plays a central role in the progression of renal diseases. This provides a basis for targeting microorganisms in the treatment of renal diseases. However, the specific mechanism requires further exploration, especially to clarify the causal effect of specific gut flora (e.g., urease-producing bacteria) on the progression of HN, and to develop precise therapeutic strategies targeting to gut flora. Indeed, medicines targeting to the uric acid transporters in intestines and kidneys, as the crucial research hotspot of urate-lowering therapy, has also been widely studied. It has been confirmed that TCMs with regulatory activity on urate transporters in the intestine and kidney, such as Dendrobium officinalis Six Nostrum, Combination of *Polyonum Cuspidatum* and *Ramulus Cinnamomi* and Tongfengting, mainly achieve this potential by inhibiting URAT1 and upregulating ABCG2.

In general, the role of intestinal therapy in HN may show dynamic changes with the disease course and the individual differences of the patients. In the early stage, or in patients with compensatory enhancement of intestinal excretion, intestinal modulation may be regarded as a core tool for HN management. However, in cases of severely impaired renal function, the combination of renal-targeted therapy and intestinal-targeted therapy is required. What cannot be ignored is that the multi-targeting properties of TCMs (especially dual gut-kidney regulation) provide a theoretical basis for treating HN as a potential alternative intervention.

### 4.2 The dilemma of TCMs from basic research to clinical application

In fact, the treatment of HN with TCMs faces many dilemmas in transitioning from basic research to clinical application, such as the complexity of Chinese medicines’ components, insufficient bioavailability, and challenges in quality control. In the future, the application of emerging technologies in the research of TCMs will provide a new technical direction for the treatment of HN with TCMs. Especially in the current fermentation processes, both single-strain and multi-strain collaborative fermentation are utilized. These methods can promote the release of effective components from TCMs and the decomposition and transformation of toxic substances, and are gradually being applied to TCM fermentation (Li Q. Y. et al., 2021; Qu et al., 2023). For example, Wang et al. used *Bacillus subtilis* to ferment Astragalus membranaceus, thereby increasing the bioavailability of Astragalus polysaccharides. Mechanically, fermented Astragalus membranaceus significantly decreases serum UA levels and increases the abundance of butyrate-producing enzymes in HUA mice (Wang R. et al., 2023). In addition to single-flavored Chinese medicine, fermentation technology can also be applied to TCM formulas (Selvaraj and Gurumurthy, 2023). Luo et al. fermented Simiao Pill with compound bacterium agent consisting of *Mucor* enzymes, *Saccharomyces cerevisiae*, and lactic acid bacteria, and selected frankincense-associated bacteria as the strain for fermenting Simiao Pill, which was then used to treat HN mice. Oral treatment with fermented Simiao Pill exhibited superior anti-hyperuricemic activity, possibly due to the release of more active substances (Luo, 2024). However, the technology of fermenting Chinese herbal medicine also has limitations, such as unclear mechanisms, a low degree of industrialization, and unstable safety, necessitating further research.

In recent years, with the development of nanotechnology, the “nanotization” of TCMs has brought new ideas for the innovative research of TCMs, improving the oral bioavailability and tissue targeting of active ingredients, thus promoting the transformation of TCMs from basic research to bedside application. For instance, exosomes or exosome-like nanovesicles derived from herbal medicines, as natural nano-preparations (Dad et al., 2021), have been confirmed to exhibit potent anti-inflammatory, antioxidant, immune-regulatory activities (Chen et al., 2021), as well as anti-inflammatory bowel disease (IBD) effects (Li D. et al., 2023). Besides, due to their small size, strong penetration and resistance to acid, alkali, and high temperature, plant-derived exosomes or exosome-like nanovesicles are ideal carriers for drug delivery (Kim et al., 2022). To address the low aqueous solubility and non-selective toxicity of Shikonin, Matias Cardoso et al. designed a self-assembled hyaluronic acid-zein nanogel loaded with Shikonin, which selectively inhibited LPS- and nigericin-induced activation of the macrophage NLPR3 inflammasome. In short, the application of nanoagents in the field of TCMs opens up new avenues for the treatment of HN. By improving the physical and chemical properties, biological activity, and targetability of TCMs, nanotechnology significantly promotes the clinical transformation of novel TCMs drugs in the future. Nevertheless, there is insufficient evidence on the safety of nanomaterials applied to humans, as well as regulatory policies, which should be given more attention.

At present, the research on the treatment of HN through the intestinal tract is still in the initial stage. Therefore, it is necessary to further strengthen the research to determine the optimal treatment strategy, including the regulation of intestinal flora, dosage, and duration of treatment, and other related issues, and to evaluate the effectiveness and safety of HN treatments through large-sample, multi-center studies, further clarifying the mechanisms by which intestinal therapy plays a role in the treatment of uric acid nephropathy. By combining genomics, metabolomics and spatial transcriptomics, we can systematically analyze the composition of intestinal flora, the targets of active ingredient, and the specific effect on multiple renal cell types modulated by TCMs. This approach helps to clarify the regulatory mechanisms of TCMs against HN. For instance, single-cell sequencing technology enables in-depth analysis of the effects of herbal medicines on specific cellular subpopulations, as well as the modulatory effect of TCMs on the multiple signaling pathways in the HN kidney. Particular attention is given to the origin of myofibroblasts and fibroblasts, such as EMT, macrophage-to-myofibroblast transition (MMT), etc. In the future, artificial intelligence will be applied to clinical data analysis to integrate multi-omics data (genomics, metabolomics, and microbiomics) with electronic health records. This integration aims to develop personalized TCMs treatment plans that optimize therapeutic efficacy and minimize adverse effects (Medicine, 2024).

Overall, TCM formulas and single herb that have a dual-regulatory effect on the intestine and kidneys in HN treatment, as a novel therapeutic approach, hold good prospects for preventing or alleviating the progression of kidney disease secondary to hyperuricemia. With the integration of multiple disciplines and the application of emerging technologies, further advancements will be achieved in the research of anti-HN TCMs in the future.
